# The Evolving Role of Intralesional Therapy in In-Transit Melanoma

**DOI:** 10.3390/curroncol33060344

**Published:** 2026-06-09

**Authors:** Celine Jeun, Mackenzie M. Mayhew, Kate Joshua, Russell G. Witt

**Affiliations:** Division of Surgical Oncology, Department of Surgery, University of Virginia, Charlottesville, VA 22908, USA; cj5um@virginia.edu (C.J.);

**Keywords:** in-transit melanoma, intralesional therapy, oncolytic immunotherapy, tumor microenvironment, immune checkpoint inhibitor, combination strategy

## Abstract

In-transit melanoma represents a biologically aggressive form of locoregional disease in which effective management requires integration of local tumor control with systemic immune engagement. Although traditional regional therapies achieve high response rates, they have not consistently translated into durable systemic survival. This review evaluates the clinical development and mechanistic rationale of intralesional therapies, including cytokine-based approaches, oncolytic viruses, immunocytokines, and energy-based delivery platforms, as immunologic intermediaries. Analysis of clinical trial data suggests that outcomes may heavily depend on an agent’s ability to induce immunogenic cell death and sustain antigen presentation. Platforms such as talimogene laherparepvec (T-VEC), vusolimogene oderparepvec (RP1), and tavokinogene telseplasmid with electroporation (Tavo-EP) demonstrate enhanced activity in combination with checkpoint blockade, whereas therapies limited to pattern-recognition receptor activation have shown inconsistent efficacy in randomized trials. Emerging noninvasive technologies, such as focused ultrasound, may further expand strategies for remodeling the immunosuppressive tumor microenvironment to enable immune sensitization. These findings support a shift toward mechanism-based treatment selection in which locoregional therapies function to overcome immune resistance rather than solely reduce tumor burden.

## Simple Summary

In-transit melanoma is an aggressive form of melanoma in which skin cancer spreads through the lymphatic system, forming clusters of tumors between the original site and nearby lymph nodes. These tumors are challenging to treat because they can be numerous and spread across large areas, often making surgery difficult. This review explores the evolution of intralesional therapies, which are treatments injected directly into tumors to destroy cancer cells while priming the immune system to attack cancer throughout the body. We examine options ranging from early immune-stimulating agents to modern oncolytic viruses, targeted proteins, and energy-based technologies like focused ultrasound. Combining these local injections with systemic immunotherapy offers a path to overcoming treatment resistance that neither approach achieves alone. These findings guide the design of future clinical trials and highlight the importance of personalized treatment sequencing to improve outcomes for patients.

## 1. Introduction

**Figure 1:**
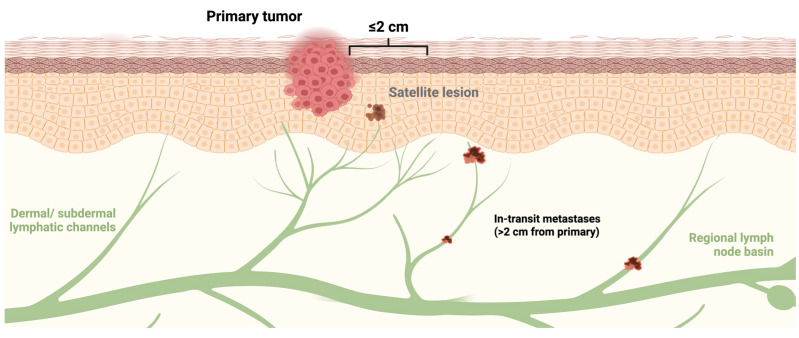
Diagram showing how melanoma cells spread through dermal lymphatic channels between the primary tumor and the regional lymph node basin. Lesions located within 2 cm of the primary site are depicted as satellite lesions, while those occurring beyond this distance are classified as in-transit metastases. Created in BioRender. Jeun, C. (2026) https://BioRender.com/5vfwbo9.

In-transit melanoma (ITM) represents a distinct and aggressive pattern of locoregional spread characterized by the entrapment and growth of tumor cells within the dermal and subdermal lymphatic channels [1,2,3]. It is defined as the presence of cutaneous or subcutaneous metastases occurring more than 2 cm from the primary melanoma site but proximal to the regional lymph node basin (Figure 1) and is classified as stage III disease (N1c –N3c) under the 8th edition of the American Joint Committee on Cancer staging system [4,5,6,7]. ITM occurs in approximately 5% to 10% of patients with cutaneous melanoma, most commonly within the first several years following the primary diagnosis [5,8,9]. While frequently observed in the extremities, ITM can also manifest in the head and neck region [6,8]. Clinically, disease burden is highly variable, ranging from isolated lesions amenable to surgical excision to diffuse involvement of an entire extremity, often precluding complete resection [7,10,11]. This clinical heterogeneity, combined with the accessibility of cutaneous disease, has made ITM uniquely amenable to locoregional therapeutic strategies, including intralesional therapies that aim to achieve both local tumor control and systemic immune activation.

The pathophysiology of ITM is incompletely understood but is predominantly attributed to intralymphatic dissemination, without widely accepted, established pathways. Disruption of lymphatic architecture may promote tumor spread through lymphostasis-driven remodeling, without changes in vascular endothelial growth factor C (VEGF-C) expression [12]. The heterogeneous recurrence patterns observed in ITM have prompted alternative explanations for locoregional spread, including angiotropism, the migration of tumor cells along the abluminal surfaces of blood and lymphatic vessels [1,13]. At the molecular level, chemokine signaling appears to play a role, as interleukin-8 (IL-8/CXCL8) expression has been associated with increased risk of ITM development [9]. These mechanisms should be interpreted as evolving models of ITM biology rather than fully established pathways.

Despite its locoregional classification, ITM reflects an aggressive tumor biology with systemic implications. Survival outcomes are strongly influenced by nodal status, with distant metastasis-free survival declining from approximately 70 months in node-negative stage IIIB disease to less than 20 months in node-positive stage IIIC disease [14,15]. Correspondingly, five-year melanoma-specific survival ranges from approximately 81% in stage IIIB N1c disease to 32% in stage IIID [4,11].

Management strategies are therefore dictated by both disease burden and underlying biology. Surgical resection remains the standard of care for limited disease, while regional therapies such as isolated limb perfusion (ILP) and isolated limb infusion (ILI) provide effective locoregional control for unresectable extremity-confined disease, with response rates approaching 77% and 64%, respectively [15,16,17]. Despite their regional efficacy, these procedures seldom improved overall survival [18,19]. Immunotherapy and targeted agents have shifted treatment goals toward achieving durable systemic control [20,21,22,23].

Within this evolving therapeutic landscape, intralesional therapy has emerged as a complementary and strategically distinct modality, particularly suited to the accessible and often multifocal nature of ITM. By enabling direct tumor targeting while simultaneously promoting systemic immune activation, intralesional therapies bridge local and systemic treatment approaches. In some patients, this immune activation may extend beyond injected lesions, producing abscopal regression at distant tumor sites. This review examines the development of intralesional therapies in melanoma, with a focus on their application in ITM, including current agents, combination strategies, and emerging platforms.

## 2. Historical Intralesional Therapy

The conceptual foundation of intralesional therapy for ITM is rooted in early observations that localized immune stimulation could effectively induce tumor regression [6]. These effects were not always confined to the injection site. Early intralesional therapies occasionally produced regression in distant, uninjected tumors, an observation consistent with an abscopal effect and suggestive of systemic immune activation distinct from direct local cytotoxicity. One of the earliest agents used was Bacillus Calmette–Guerin (BCG), an attenuated mycobacterial vaccine that contributed to the early development of local immunotherapy in melanoma [24]. However, pooled analyses of clinical trials demonstrated a more modest overall complete response (CR) rate of approximately 19%, and clinical adoption declined because of severe systemic toxicities, including disseminated intravascular coagulation and anaphylaxis [25,26]. Other early approaches utilized potent contact sensitizers like dinitrochlorobenzene (DNCB). While DNCB produced lesion response rates of 60% to 90%, it was largely abandoned due to its mutagenic potential [5,6].

Intralesional cytokine therapy subsequently emerged as a more targeted immunologic strategy. Unlike systemic high-dose interleukin-2 (IL-2), which is limited by profound toxicities such as capillary leak syndrome, intralesional administration delivers high local concentrations within the tumor microenvironment (TME) while minimizing systemic exposure [6,26,27]. A systematic review of six observational trials encompassing over 2000 lesions reported lesion-level and patient-level CR rates of 78% and 50%, respectively [28]. Adverse events were largely limited to localized discomfort and transient flu-like symptoms [29,30]. Intralesional IL-2 provided early clinical evidence that localized immune activation could induce tumor regression, laying the groundwork for the development of contemporary intralesional immunotherapies.

## 3. Contemporary and Future Intralesional Therapies

The agents discussed in this section represent distinct mechanistic classes, spanning oncolytic viruses, cytokines, chemoablative compounds, and electroporation-based gene delivery approaches (Table 1).

### 3.1. T-VEC

Talimogene laherparepvec (T-VEC) is the first and only oncolytic viral immunotherapy approved by both the U.S. Food and Drug Administration (FDA) and the European Medicines Agency (EMA) for the treatment of unresectable cutaneous, subcutaneous, and nodal melanoma metastases in adults [24,31]. Engineered from a modified herpes simplex virus type 1 (HSV-1), T-VEC is understood to operate through a dual mechanism that distinguishes it from conventional cytotoxic agents [32,33]. At the tumor level, the virus selectively replicates within malignant cells, ultimately inducing direct tumor cell lysis and the release of tumor-associated antigens [6,31,34]. By also encoding granulocyte-macrophage colony-stimulating factor (GM-CSF), T-VEC recruits and matures dendritic cells at the injection site [11,35]. By this mechanism, these effects make T-VEC an intratumoral priming agent with the potential to drive immune responses beyond the injected lesions [36,37].

The clinical efficacy of T-VEC was established in the phase III OPTiM trial, which randomized 436 patients with unresectable stage IIIB-IV melanoma to intralesional T-VEC or subcutaneous GM-CSF. The primary endpoint, durable response rates (response lasting ≥6 months), was significantly higher with T-VEC (16.3% vs. 2.1%; *p* < 0.001), with a superior overall response rate (ORR) as well (26.4% vs. 5.7%). Responses occurred in both injected and uninjected lesions, including distant visceral lesions, consistent with systemic immune activation induced by intratumoral therapy. The benefit was most pronounced in stage IIIB-IVM1a patients without visceral metastases, who achieved an ORR of 40.5% vs. 2.3% and a median overall survival (OS) of 41.1 vs. 21.5 months with T-VEC vs. GM-CSF, respectively (hazard ratio (HR) 0.57; 95% confidence interval (CI) 0.40–0.80). T-VEC maintained a favorable safety profile, with predominantly grade 1–2 flu-like symptoms and injection-site reactions [36]. Severe grade 3–4 events, such as cellulitis, are rare, making it a well-tolerated option for patients who may not be candidates for intensive systemic therapy [33,34]. These data established T-VEC as an effective locoregional therapy, a finding subsequently corroborated across real-world institutional series.

Since its approval, single- and multi-institutional studies have reported CR rates between 39% and 62% [32,37,38]. These results exceed the 10.8% CR rate reported in the original OPTiM trial [36]. Tumor diameter, lesion count, and metastasis type have emerged as predictors of treatment success, with cutaneous lesions demonstrating higher response rates than nodal disease [32]. Efficacy appears consistent across patient age groups and prior treatment history, with CR rates of 37% reported even in patients who previously failed systemic checkpoint inhibition [34,39]. Data on immune checkpoint inhibitors (ICIs) as monotherapy specifically for ITM are limited, as locoregional disease was underrepresented in the trials that established checkpoint inhibition in the advanced and adjuvant settings [13,31]. A recent international head-to-head analysis demonstrated that T-VEC nearly doubled the odds of achieving CR compared with ICIs used as first-line therapy (odds ratio (OR) 1.96) [31], although this comparison was limited by its retrospective nature and likely selection bias, as the T-VEC cohort included patients with more favorable disease features, including a higher proportion with stage IIIB disease.

While systemic ICIs remain the standard of care for distant disease, T-VEC holds a unique niche for ITM as a bridge or adjunct to systemic therapies [35,40,41]. T-VEC’s success as a locoregional immunostimulant has validated intratumoral delivery as a platform for coordinating local tumor destruction with systemic immune engagement [6,36,42]. This functional framework has paved the way for a growing pipeline of complementary agents that exploit distinct mechanistic pathways to further define the role of intralesional therapy in the modern era [5,6,11].

While T-VEC has established efficacy in the unresectable and metastatic settings, recent data highlight its expanding role in the neoadjuvant management of advanced melanoma. A randomized, open-label, phase II trial evaluated six doses of neoadjuvant T-VEC followed by surgery versus upfront surgery alone in 150 patients with resectable stage IIIB-IVM1a disease [43,44]. Initial analysis demonstrated improved 2-year recurrence-free survival (RFS) (29.5% vs. 16.5%; HR 0.75) alongside a pathologic complete response (pCR) rate of 17.1% in the neoadjuvant T-VEC cohort [43]. Long-term follow-up further demonstrated durable improvements in RFS and OS [44]. These findings suggest that the locoregional tumor destruction and systemic immune priming induced by T-VEC can translate into durable clinical benefit when administered prior to surgical resection.

### 3.2. Intralesional IL-2

Although intralesional IL-2 predates T-VEC as a clinical tool, its immunologic role has become better understood in recent years [11,26,31]. High-dose intravenous IL-2 can produce durable remissions with OS reaching nearly 65 months in responders, yet its use remains largely constrained to select specialized centers [45]. While preserving the high response rates established in earlier systematic reviews, notably a 78% lesion-level CR, contemporary institutional series have further corroborated these findings with ORRs exceeding 80% and pCR rates reaching up to 51% [26,28,29]. A retrospective analysis demonstrated that intralesional IL-2 yields progression-free survival (PFS) and OS rates comparable to T-VEC in unresectable stage III melanoma [46]. The ease of use is particularly relevant for elderly patients or those with significant comorbidities who are poor candidates for more toxic regimens or invasive regional therapies like ILP [29,35]. Unlike T-VEC, which requires stringent biosafety level 1 protocols and specialized handling due to its viral nature, intralesional IL-2 is a cytokine-based therapy and therefore does not carry a risk of viral transmission [46].

More recent studies suggest that intralesional IL-2 may extend beyond local tumor ablation to promote broader immune engagement [6,26]. Immunohistochemical analysis of post-treatment biopsies suggests this systemic activity is associated with, and potentially mediated by, CD8+ T-cell infiltration, with responders demonstrating significantly greater intratumoral lymphocyte density than nonresponders [26,47]. Up to 37% of patients in stage IV cohorts exhibit responses in distant metastases, though IL-2 appears most effective in patients whose disease is primarily locoregional at treatment initiation [27,45,47]. Long-term data suggest that the durability of response with IL-2 compares favorably with regional chemotherapy, with nearly two-thirds of initial complete responders achieving sustained disease control [28,30]. Consequently, while newer agents continue to be developed, intralesional IL-2 remains a possible locoregional option in appropriately selected patients [30,48].

### 3.3. Darleukin and Daromun

To build upon the efficacy of standard intralesional IL-2 while enhancing tumor retention, researchers developed targeted immunocytokines such as Darleukin (L19IL2) [49]. The L19 antibody targets the extra-domain B (EDB) of fibronectin, a marker of tumor neovasculature, enabling preferential localization of the cytokine fusion protein within the TME [49,50,51]. By anchoring to the tumor stroma, this fusion protein extends cytokine residence time to over 72 h compared with the <24 h retention seen with non-targeted cytokines [50].

The pharmacological success of the L19 platform led to the development of Daromun, a dual-cytokine formulation that combines L19IL2 with L19TNF [50,52]. This combination exploits the synergy between IL-2-mediated T-cell recruitment and tumor necrosis factor (TNF)-induced vascular permeability, which facilitates deeper immune infiltration into the TME [49]. The PIVOTAL phase III trial, which evaluated neoadjuvant intralesional daromun followed by surgery against upfront surgery alone in patients with resectable stage IIIB/C melanoma, validated this approach. The Daromun cohort achieved a 21% pCR and demonstrated significantly improved recurrence-free survival (HR 0.59; *p* = 0.005) [50]. Additional evaluation of Daromun is ongoing in the Neo-DREAM trial (NCT03567889) [52]. These results emphasize the potential of EDB-targeted immunocytokines to effectively prime the host immune system and address micrometastatic disease prior to surgical resection.

### 3.4. PV-10

While targeted cytokines focus on direct immune modulation, a parallel approach to intralesional therapy utilizes direct chemoablation to trigger secondary immune recruitment [19,35]. A notable agent in this category is PV-10, a solution of rose bengal disodium, a water-soluble xanthene dye [6]. Unlike viral or cytokine-based platforms, PV-10 operates through lysosomal lysis [11,37]. This necrotic cascade promotes tumor antigen release along with damage-associated signals such as high mobility group box 1 (HMGB1), which activate dendritic cells and induce a tumor-specific T-cell response. Preclinical models and clinical observations further demonstrate increased tumor antigen-specific CD8+ T-cell activity in peripheral compartments, consistent with systemic immune activation following intralesional therapy [53].

In a Phase II cohort of 80 patients with refractory melanoma, PV-10 produced an ORR of 51% and a CR rate of 26% in target lesions. Notably, the CR rate increased to 50% in a subgroup analysis where all clinically evident disease was injected, highlighting the importance of comprehensive lesion treatment [54]. A propensity score-matched comparison demonstrated that survival outcomes with PV-10 were not significantly different from ILI, but with a safety profile consisting primarily of self-limited, grade 1–2 injection-site reactions [19]. A subsequent institutional series confirmed PV-10’s efficacy with ITM, achieving a 46% CR rate by 8 weeks with toxicity largely limited to transient, localized pain [55]. Despite encouraging Phase II activity, PV-10 remains without regulatory approval [17]. The subsequent Phase III trial (NCT02288897) was terminated early for insufficient enrollment, leaving its place in the treatment landscape unresolved [56].

### 3.5. LTX-315

While PV-10 achieves immune activation as a downstream consequence of non-selective chemoablation, a newer class of agents couples targeted cytotoxicity with defined immunogenic cascades to more deliberately reprogram the TME [57]. The oncolytic peptide LTX-315 triggers the disintegration of cytoplasmic organelles, specifically mitochondria, to release potent immunogenic signals [6,58]. This cytotoxic cascade depletes immunosuppressive populations while expanding polyfunctional cytotoxic T cells, suggesting the potential to reverse local immune exclusion [59]. In Phase I trials, LTX-315 demonstrated partial and complete regression in 28% of injected tumors and stable disease in 58% of patients. Biopsies of injected lesions have also revealed a significant 2- to 30-fold increase in CD8+ T-cell infiltration following treatment [6]. This capacity to overcome local immune resistance makes LTX-315 a promising candidate for synergistic combination with systemic ICIs [57,59].

### 3.6. Tavo

Among the most mechanistically distinct entries in the emerging intralesional pipeline is tavokinogene telseplasmid (Tavo), a DNA plasmid encoding human IL-12 delivered via in vivo electroporation [60,61]. Brief electrical pulses permeabilize tumor membranes to facilitate plasmid uptake. This localized IL-12 expression avoids the dose-limiting systemic toxicities typical of recombinant cytokine therapy [62]. IL-12 acts on multiple immune effectors, augmenting natural killer (NK) cell and T-cell activity, upregulating interferon gamma, and enhancing antigen presentation while also reducing immunosuppressive regulatory T cells within the TME [63,64]. In a Phase II study, Tavo with electroporation produced an ORR of 35.7% in patients with stage III/IV melanoma [64]. Abscopal activity was substantial, with disease contraction in non-targeted lesions observed in nearly half of evaluable patients [63,65].

### 3.7. RP1

Earlier pattern-recognition receptor (PRR)-based approaches, including toll-like receptor 9 (TLR-9) agonists such as SD-101, tilsotolimod, and CMP-001, demonstrated mechanistic promise but failed to produce single-agent efficacy sufficient to establish a defined treatment role [66,67,68,69,70]. This evolution is best exemplified by vusolimogene oderparepvec (RP1), a second-generation HSV-1-based oncolytic immunotherapy that retains the GM-CSF expression scaffold of T-VEC while incorporating an additional fusogenic protein, gibbon ape leukemia virus glycoprotein receptor-deficient (GALV-GP-R) [71,72]. This protein drives infected tumor cells to fuse into syncytia, substantially amplifying tumor cell destruction and antigen exposure [71].

Recent evidence suggests that RP1 activates stimulator of interferon genes (STING)-mediated interferon signaling in immune cells, which may alter the TME and upregulate programmed death-ligand 1 (PD-L1) expression to sensitize tumors to systemic checkpoint inhibition via programmed cell death protein 1 (PD-1) [73]. This is evidenced by registrational Phase II data from the IGNYTE trial in anti-PD-1-failed melanoma, where the combination of RP1 and nivolumab reached a confirmed ORR of 32.9% with a 15.0% CR rate. This shift from monotherapy to a synergistic prime-boost strategy is now the subject of the global Phase III IGNYTE-3 trial, evaluating the regimen in the difficult-to-treat post-immunotherapy setting [72]. However, despite encouraging Phase II activity, the FDA subsequently issued a complete response letter for the RP1 and nivolumab biologics license application, highlighting ongoing uncertainty regarding whether the current IGNYTE dataset was sufficient to support approval [74].

**Figure 2:**
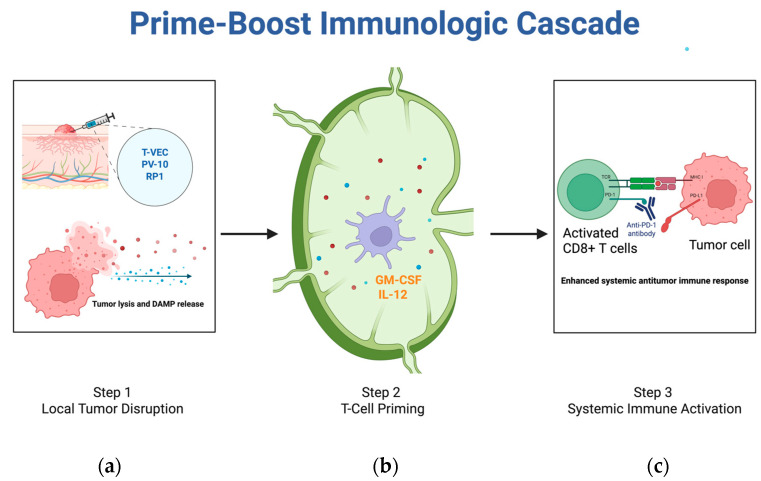
Schematic representation of the three-stage process of in situ vaccination. (**a**) Step 1: Initial treatment leads to localized tumor breakdown using agents like T-VEC or RP1, which trigger the release of cell-death signals and tumor-specific antigens (represented by the red and blue dots). (**b**) Step 2: These signals migrate to the lymph nodes, where cytokines like IL-12 and GM-CSF facilitate the priming and maturation of T cells. (**c**) Step 3: These newly activated CD8+ T cells circulate through the bloodstream to identify and eliminate cancerous cells throughout the body, a process that can be further amplified when paired with systemic checkpoint inhibitors. Horizontal arrows indicate the sequential, stepwise progression of the immunologic cascade from local intervention to systemic antitumor immunity. Created in BioRender. Jeun, C. (2026) https://BioRender.com/jcfu8x2.

These developments show a shift toward rational combination strategies that integrate local tumor disruption with systemic immune modulation (Figure 2).

## 4. Intralesional Combination Strategies

To leverage immune priming and local control effects of intralesional therapy, intralesional trials have moved toward integrated protocols that use locoregional tumor disruption to overcome resistance to systemic monotherapy (Table 2) [47,62]. Currently, these combination strategies fall into two primary mechanistic categories: intralesional priming with systemic checkpoint blockade and local ablative or immune-modulatory approaches combined with topical agents.

### 4.1. Intralesional Agents Combined with Immune Checkpoint Inhibitors

T-VEC’s capacity for in situ antigen presentation and dendritic cell recruitment provides a mechanistically compelling basis for combination with systemic ICIs [39,42]. A randomized phase II trial evaluating T-VEC in combination with ipilimumab demonstrated a significantly higher ORR compared to ipilimumab alone (35.7% vs. 16.0%), with greater antitumor activity observed without additional toxicity or new safety signals [75]. The phase III MASTERKEY-265 trial evaluated T-VEC with pembrolizumab, yielding a numerically higher ORR (48.6% vs. 41.3%) but failing to meet its primary endpoints of improved PFS or OS in the overall population [76]. The lack of benefit may reflect multiple factors related to study design and patient selection, including disease heterogeneity, prior treatment exposure, and the inclusion of patients with widespread visceral disease, among whom T-VEC’s locoregional mechanism may be less clinically impactful. [32,34,36,38].

Further support for this interpretation comes from the phase II MASTERKEY-115 trial. Among 71 patients with PD-1-refractory advanced melanoma, T-VEC plus pembrolizumab produced markedly higher response rates in those whose disease recurred on or after adjuvant anti-PD-1 therapy (ORR 40.0–46.7%) than in those who progressed on anti-PD-1 in the metastatic setting (ORR 0–6.7%), with a manageable safety profile. The higher injected-to-uninjected lesion ratio and predominance of stage IIIB–IVM1a disease in the adjuvant recurrence cohorts reinforce that T-VEC’s activity may be most pronounced when a high proportion of metastatic lesions are accessible for direct injection [77]. The neoadjuvant NIVEC trial extends this principle to earlier-stage disease. Intralesional T-VEC plus nivolumab for resectable early metastatic melanoma yielded a 65% major pathological response rate and a 75% 1-year event-free survival, with grade 3 treatment-related adverse events occurring in only 8% of patients [78]. These data identify a population of patients with locoregionally confined, injectable disease in whom T-VEC-checkpoint inhibitor combinations retain meaningful clinical activity despite the negative overall results of MASTERKEY-265.

Tavo-EP with pembrolizumab was explored specifically to address melanomas with low baseline T-cell infiltration [62,63]. In combination with pembrolizumab, Tavo-EP has shown activity in PD-1-refractory, poorly inflamed tumors, producing durable responses without the systemic toxicity of recombinant IL-12 [64]. Preliminary Phase Ib data for PV-10 combined with pembrolizumab in patients with stage IV melanoma demonstrated a 9% CR rate and a 65% ORR [55]. Separately, retrospective studies suggest that intralesional IL-2 can salvage patients failing PD-1 monotherapy, boosting locoregional control and PFS [46,47].

Not all combinations have proven synergistic. The ILLUMINATE-301 phase III trial found no significant improvement in ORR when intratumoral tilsotolimod was added to ipilimumab [79]. These findings suggest that effective combination strategies may depend on therapies capable of inducing robust immunogenic cell death and durable immune activation, rather than pattern-recognition signaling alone, a principle that helps explain the divergent clinical trajectories of viral and cytokine-based platforms versus TLR agonists [58,73,79].

### 4.2. Intralesional Therapy Combined with Topical Immune Modulators

In addition to systemic combinations, intralesional therapies may also be paired with topical agents that modulate the local TME. Topical imiquimod, a TLR-7/8 agonist, combined with intralesional T-VEC or IL-2 has demonstrated efficacy for patients with extensive, superficially distributed ITM and contraindications to systemic or regional therapy [5,6,33]. Case series have reported high CR rates and excellent tolerability with T-VEC plus imiquimod, and the combination with IL-2 has been reported to control mixed cutaneous and subcutaneous disease while slowing the emergence of new lesions [6,33]. Though evidence remains largely observational, these combinations represent a clinically accessible option for appropriately selected patients [33,48].

## 5. Alternative and Emerging Locoregional Therapies

The landscape of ITM management is expanding beyond purely pharmacologic agents to incorporate emerging technologies that reshape the TME through physical rather than chemical disruption [80,81,82,83,84]. Earlier innovations in electroporation-mediated cytokine delivery established that technical platforms could meaningfully enhance drug uptake and local immune engagement [62,64]. Building on this precedent, a new generation of energy-based modalities aims to drive antitumor immune responses through direct tissue manipulation alone [82].

### 5.1. Electrochemotherapy

Electrochemotherapy (ECT) combines low-dose cytotoxic agents with brief high-voltage electrical pulses that transiently permeabilize tumor cell membranes, enhancing intracellular drug uptake while limiting systemic exposure [85,86,87]. Meta-analyses in metastatic cutaneous melanoma report ORRs of approximately 75–80% and CR rates of 35–50% at the patient level, with pooled lesion-level response and CR rates of 77.0% and 53.5%, respectively [88,89]. In locally advanced stage III/IV melanoma with in-transit involvement, reported CR rates range from 24% to 85.7%, with ORRs of 63% to 100%, reflecting variability in lesion selection and treatment protocols [90]. Beyond cytoreduction, ECT induces immunogenic cell death through the release of damage-associated molecular patterns (DAMPs), including calreticulin, adenosine triphosphate (ATP), and HMGB1, with the potential to promote dendritic cell activation and adaptive antitumor immunity [87,91].

Combining electrochemotherapy with pembrolizumab matches ECT’s local efficacy while significantly boosting systemic PFS, suggesting that ECT-induced cell death may amplify the effects of checkpoint inhibition [87,92]. Clinical outcomes may depend less on the potency of individual agents and more on how effectively local immune priming is combined with systemic checkpoint inhibition [37,93]. Optimizing these combinations will require prospective biomarker integration, particularly baseline tumor inflammation status and prior immunotherapy exposure, to match patients to regimens most likely to remodel rather than merely stimulate the TME [35,41].

### 5.2. Focused Ultrasound

Focused ultrasound (FUS) represents a noninvasive, energy-based approach to locoregional therapy [81,84]. By focusing acoustic energy at a precise focal point, FUS can induce thermal ablation, mechanical disruption, or subablative bioeffects depending on delivery parameters [84,94,95]. These effects have been demonstrated across multiple FUS modalities, including thermal and mechanical approaches, as well as related techniques such as boiling histotripsy, which induces cavitation-mediated tissue disruption [81,95]. This tunability is particularly relevant in ITM, where lesions vary significantly in depth and anatomical accessibility [35].

At ablative intensities, FUS produces coagulative necrosis with release of tumor antigens and damage-associated signals [84,96]. In contrast, subablative exposures may modulate tissue architecture and vascular dynamics, potentially favoring immune cell infiltration without requiring extensive tissue necrosis [81,82]. Preclinical data suggest these complementary effects may enhance responsiveness to systemic checkpoint inhibition, positioning FUS as an immune-sensitizing adjunct rather than a purely cytoreductive modality [80,83,97].

Beyond its biological effects, FUS offers distinct practical advantages. As a versatile noninvasive modality, FUS can bypass the delivery constraints of needle-based approaches for hard-to-reach lesions while also serving as a powerful adjunct to enhance the efficacy of targeted intratumoral injections [94,97,98]. The clinical utility of this approach in ITM is currently being evaluated in a prospective trial (NCT06472661) combining FUS ablation with intratumoral polyICLC, a TLR-3 agonist, with correlative endpoints including peripheral immune profiling and tissue-level analysis [99]. In this context, FUS-mediated ablation may provide the structural disruption of the immunosuppressive microenvironment that TLR agonists alone have been insufficient to achieve, potentially rescuing the mechanistic promise of pattern-recognition signaling when paired with upstream physical priming [81,82]. If validated clinically, FUS may represent a technological interface linking local tumor ablation with systemic immune activation, aligning with emerging coordinated local-systemic treatment paradigms [81,83].

### 5.3. Regional Vascular Therapies as Alternatives

While intralesional combination strategies use localized tumor-directed therapy to promote systemic immune priming, they exist alongside regional vascular therapies that historically served as benchmarks for locoregional control in unresectable ITM. Sequencing regional melphalan-based ILI with systemic CTLA-4 blockade initially demonstrated the potential for chemoimmunotherapy synergy, yielding an ORR of 85% and a CR rate of 62% at three months, though at the cost of substantial grade 3–4 immune-related toxicity (38%) [100]. To improve this safety profile, more recent approaches have incorporated PD-1-directed inhibition. The randomized phase Ib/II NivoILP trial demonstrated that a single dose of nivolumab administered prior to ILP improved three-month CR rates (75% vs. 60% with placebo) and one-year local progression-free survival (86% vs. 67%) without increased grade 4–5 toxicity [101,102,103].

Therapeutic timing carries important clinical implications in this context. Multicenter analyses indicate that patients undergoing ILP after failing prior systemic immunotherapy achieve markedly lower CR rates (6% vs. 47%) and worse OS compared to immunotherapy-naive patients [18,35]. This suggests that prior checkpoint failure may select for immunoresistant clones less amenable to regionally induced immune priming. These findings suggest that regional therapies are most effective when deployed upfront, even if they retain activity when used as salvage after systemic failure [16,31,104]. Although ILP and ILI remain highly effective for acute locoregional tumor debulking, contemporary intralesional combination platforms are increasingly favored for their ability to coordinate local tumor control with systemic immune engagement without requiring invasive vascular isolation procedures.

## 6. Conclusions

Intralesional therapy has an expanding role in the management of in-transit melanoma, particularly in combination with systemic therapy. T-VEC remains the most established intralesional agent for unresectable locoregional disease, but newer approaches, including RP1 with nivolumab, Tavo-EP, Daromun, and PV-10, reflect a rapidly evolving field. As systemic therapy has improved, the purpose of locoregional treatment has also changed. Earlier intralesional therapies were largely judged by their ability to eradicate injected disease. More recent strategies aim to use the tumor itself as a site of immune priming, with the goal of generating responses beyond the injected lesions. This shift has influenced trial design, combination approaches, and how treatment success is measured.

The available evidence also suggests that the mechanism matters when selecting agents for combination therapy. Intralesional therapies that remodel the tumor microenvironment and support adaptive immune responses, including oncolytic viruses, cytokine-based platforms, and electroporation-based delivery, appear more suitable for combination with checkpoint inhibition than agents that rely on pattern-recognition receptor stimulation alone. The negative randomized experience with TLR9 agonists, contrasted with the clinical activity seen with T-VEC, RP1, and Tavo-EP-based combinations, suggests that effective immune priming may require broader changes within the tumor microenvironment. This concept should guide future combination strategies and agent selection.

Further work is needed to define the best timing, sequencing, and patient populations for these approaches. Earlier use of locoregional immune priming, combined with biomarkers of tumor inflammation and resistance, may help identify patients most likely to benefit and maximize the systemic impact of intralesional therapy.

## Figures and Tables

**Table 1:** Intralesional agents grouped by therapeutic class with corresponding mechanisms of action and downstream immune effects in the tumor microenvironment.

Class	Agents	Mechanism	Immune Effect
Oncolytic	T-VEC	Selective viral replication and direct tumor cell lysis	Dendritic cell recruitment and maturation via GM-CSF
	RP1	HSV-1 platform using fusogenic protein to drive tumor syncytia	STING-mediated interferon signaling and PD-L1 upregulation
Cytokine	IL-2	High-dose local concentration within the TME	Systemic engagement driven by increased CD8+ T-cell infiltration
	Daromun	Dual-cytokine targeting EDB-fibronectin in neovasculature	Synergistic T-cell recruitment and enhanced vascular permeability
Chemoablative	PV-10	Acute necrotic cascade via lysosomal lysis	Release of tumor antigens and DAMPs
Platform	Tavo-EP	Electroporation-mediated delivery of IL-12 DNA plasmid	Augmented NK/T-cell activity and reduced regulatory T cells
	ECT	Electroporation of low-dose chemo	DAMP release and adaptive immunity
	FUS	Noninvasive acoustic energy inducing thermal or mechanical disruption	Release of antigens/DAMPs and modulation of tissue architecture

CD8+ (CD8-positive T lymphocytes); DAMPs (damage-associated molecular patterns); Daromun (combination of L19IL2 and L19TNF); EDB-fibronectin (extra-domain B of fibronectin); FUS (focused ultrasound); GM-CSF (granulocyte-macrophage colony-stimulating factor); HSV-1 (herpes simplex virus type 1); IL-2 (interleukin-2); IL-12 (interleukin-12); NK (natural killer); PD-L1 (programmed death-ligand 1); PV-10 (rose bengal disodium); RP1 (vusolimogene oderparepvec); STING (stimulator of interferon genes); Tavo (tavokinogene telseplasmid); TME (tumor microenvironment); T-VEC (talimogene laherparepvec).

**Table 2:** Key clinical trials and outcomes of intralesional and combination therapies in melanoma.

Agent	Clinical Trial (s)	Primary Outcomes and Status
T-VEC	OPTiM (Phase III)	Established T-VEC as a standard locoregional therapy for unresectable melanoma
T-VEC + Pembrolizumab	MASTERKEY-265 (Phase III) MASTERKEY-115 (Phase II)	Primary endpoints (PFS, OS) not met in the overall population. Higher responses in adjuvant recurrence cohorts
T-VEC + Nivolumab	NIVEC (Phase II)	High pathologic response rates with acceptable toxicity
Daromun	PIVOTAL (Phase III)	Neoadjuvant intralesional therapy followed by surgery vs. surgery alone; improved recurrence-free survival
PV-10	NCT02288897 (Phase III)	Terminated early due to insufficient enrollment
LTX-315	Phase I Trials	Expanded cytotoxic T cells and increased intratumoral CD8+ infiltration
Tavo-EP	Phase II Study	Demonstrated locoregional and abscopal activity in stage III/IV melanoma
RP1 + Nivolumab	IGNYTE (Phase II); IGNYTE-3 (Phase III)	IGNYTE: active in anti-PD-1-failed melanoma; accelerated approval stalled by FDA CRL regarding Phase II data sufficiency IGNYTE-3: ongoing in the post-immunotherapy setting
Tilsotolimod + Ipilimumab	ILLUMINATE-301 (Phase III)	No significant improvement over ipilimumab alone
Nivolumab + ILP	NivoILP (Phase Ib/II)	Nivolumab prior to ILP improved local PFS without increased toxicity
FUS + polyICLC	NCT06472661	Ongoing; evaluating focused ultrasound ablation with intratumoral TLR-3 agonist

CD8+ (CD8-positive T lymphocytes); Daromun (combination of L19IL2 and L19TNF); FUS (focused ultrasound); IL-2 (interleukin-2); IL-12 (interleukin-12); ILP (isolated limb perfusion); LTX-315 (oncolytic peptide); OS (overall survival); PD-1 (programmed cell death protein 1); PFS (progression-free survival); polyICLC (polyinosinic-polycytidylic acid stabilized with poly-L-lysine and carboxymethylcellulose); PV-10 (rose bengal disodium); RP1 (vusolimogene oderparepvec); Tavo-EP (tavokinogene telseplasmid); TLR-3 (toll-like receptor 3); T-VEC (talimogene laherparepvec).

## Data Availability

No new data were created or analyzed in this study.

## Abbreviations

The following abbreviations are used in this manuscript:

ATP	Adenosine triphosphate
BCG	Bacillus Calmette–Guerin
CI	Confidence interval
CR	Complete response
CTLA-4	Cytotoxic T-lymphocyte-associated protein 4
DAMPs	Damage-associated molecular patterns
DNCB	Dinitrochlorobenzene
ECT	Electrochemotherapy
EDB	Extra-domain B
EMA	European Medicines Agency
FDA	U.S. Food and Drug Administration
FUS	Focused ultrasound
GALV-GP-R-	Gibbon ape leukemia virus glycoprotein receptor-deficient
GM-CSF	Granulocyte-macrophage colony-stimulating factor
HMGB1	High mobility group box 1
HR	Hazard ratio
HSV-1	Herpes simplex virus type 1
ICI(s)	Immune checkpoint inhibitor(s)
IL	Interleukin
IL-8/CXCL8	Interleukin-8
ILI	Isolated limb infusion
ILP	Isolated limb perfusion
ITM	In-transit melanoma
NK	Natural killer (cells)
OR	Odds ratio
ORR	Overall response rate
OS	Overall survival
pCR	Pathologic complete response
PD-1	Programmed cell death protein 1
PD-L1	Programmed death-ligand 1
PFS	Progression-free survival
polyICLC	Polyinosinic-polycytidylic acid stabilized with poly-L-lysine and carboxymethylcellulose
PRR	Pattern-recognition receptor
RFS	Recurrence-free survival
RP1	Vusolimogene oderparepvec
STING	Stimulator of interferon genes
T-VEC	Talimogene laherparepvec
Tavo/Tavo-EP	Tavokinogene telseplasmid (electroporation)
TLR	Toll-like receptor
TME	Tumor microenvironment
TNF	Tumor necrosis factor
VEGF-C	Vascular endothelial growth factor C
